# Health effects of adopting low greenhouse gas emission diets in the UK

**DOI:** 10.1136/bmjopen-2014-007364

**Published:** 2015-04-28

**Authors:** James Milner, Rosemary Green, Alan D Dangour, Andy Haines, Zaid Chalabi, Joseph Spadaro, Anil Markandya, Paul Wilkinson

**Affiliations:** 1 Department of Social and Environmental Health Research, London School of Hygiene and Tropical Medicine, London, UK; 2 Department of Population Health, London School of Hygiene and Tropical Medicine, London, UK; 3 Leverhulme Centre for Integrative Research on Agriculture and Health, London, UK; 4 Basque Centre for Climate Change, Bilbao Bizkaia, Spain

**Keywords:** EPIDEMIOLOGY

## Abstract

**Objective:**

Dietary changes which improve health are also likely to be beneficial for the environment by reducing emissions of greenhouse gases (GHG). However, previous analyses have not accounted for the potential acceptability of low GHG diets to the general public. This study attempted to quantify the health effects associated with adopting low GHG emission diets in the UK.

**Design:**

Epidemiological modelling study.

**Setting:**

UK.

**Participants:**

UK population.

**Intervention:**

Adoption of diets optimised to achieve the WHO nutritional recommendations and reduce GHG emissions while remaining as close as possible to existing dietary patterns.

**Main outcome:**

Changes in years of life lost due to coronary heart disease, stroke, several cancers and type II diabetes, quantified using life tables.

**Results:**

If the average UK dietary intake were optimised to comply with the WHO recommendations, we estimate an incidental reduction of 17% in GHG emissions. Such a dietary pattern would be broadly similar to the current UK average. Our model suggests that it would save almost 7 million years of life lost prematurely in the UK over the next 30 years and increase average life expectancy by over 8 months. Diets that result in additional GHG emission reductions could achieve further net health benefits. For emission reductions greater than 40%, improvements in some health outcomes may decrease and acceptability will diminish.

**Conclusions:**

There are large potential benefits to health from adopting diets with lower associated GHG emissions in the UK. Most of these benefits can be achieved without drastic changes to existing dietary patterns. However, to reduce emissions by more than 40%, major dietary changes that limit both acceptability and the benefits to health are required.

**Strengths and limitations of this study**

- This paper presents a novel application of an optimisation approach used to estimate dietary changes in the UK which would meet nutritional and climate change mitigation targets.
- The method generates dietary patterns at a greater level of detail than previous assessments.
- The work also accounts for the potential acceptability of the modelled diets through the use of data on consumer behaviour.
- The resulting impacts on mortality in the UK have been modelled using disease-specific time lag curves.
- The limitations of the study mostly relate to inadequacies of the available data on food consumption and greenhouse gas emissions related to the diet.

## Introduction

Meeting targets for reducing greenhouse gas (GHG) emissions in many countries is likely to require significant changes to diets.^1^ ^2^ It is now relatively well established that dietary changes that reduce GHG emissions are also likely to be desirable from the standpoint of their nutritional content^3^ and health outcomes.^4^ ^5^ Previous work has modelled the potential beneficial effects of broad and often radical dietary changes on health and GHG emissions, typically based on greatly reduced consumption of animal source foods and higher consumption of fruit and vegetables.^4^ ^6–10^ However, there is a need for more detailed information on the specific composition of healthy and low-emission diets, which will help to prioritise policy action and interventions aimed at promoting healthier eating.^11^ Further, given the possible public resistance, it is important to know how radical dietary changes need to be to achieve health and climate change mitigation targets.

The recent WHO Global Burden of Disease (GBD) estimates suggest that dietary risk factors account for a tenth of the global disease burden.^12^ In the UK, current average diets fail to meet multiple UK dietary recommendations and do not contain the recommended daily amounts of many micronutrients.^13^ Diet-related ill health in the UK is estimated to cost the National Health Service (NHS) around £6 billion annually.^14^ Little work has been carried out on defining realistic diets for the UK population that are good for health while reducing GHG emissions. A recent estimate has suggested that increasing the likely acceptability of the changed diets to the UK population reduces the level of emissions reduction that could be achieved by almost two-thirds.^7^

We present here work that aims to balance improving health, reducing GHG emissions and maintaining realistic diets in the UK, finding a point at which the combined benefits are maximised. It is designed to quantify the potential health benefits of a range of dietary changes that would help meet the UK's commitment to reduce GHG emissions. We have constrained the modelled diets to be as close as possible to current food consumption, thus increasing the likelihood of acceptability. This paper provides estimates of health effects, whereas the GHG emissions resulting from the dietary changes are presented elsewhere.^15^

## Methods

We modelled a range of ‘optimised’ modifications to the average UK dietary pattern to achieve compliance with the WHO nutritional recommendations and GHG emission targets. The potential health impacts which would result from adopting the new optimised dietary patterns were estimated using life tables. The methods used to model the diets are summarised below but described in greater detail elsewhere.^15^

### Dietary data

Information on current average diets in the UK was obtained from the National Diet and Nutrition Survey (NDNS), based on 4-day food diaries for 1571 adults. All foods in the survey were included in the model. The data were aggregated to calculate average daily consumption of 42 representative (compositionally similar) food groups for males and females. The data were also used to obtain the average nutritional contents for each food group.

### Greenhouse gas emissions data

GHG emissions associated with each food group were estimated using a Life Cycle Inventory (LCI) compiled from the published literature.^2^ ^6^ ^16–21^ Where possible, we included information on food losses from production, handling, sales, cooking and consumer waste. Where LCI emissions were not available, we based our estimates on information on representative items contained within that group.

### Optimisation of future diets

We used an optimisation method to generate modified average dietary intake patterns for the UK which met the WHO nutritional recommendations.^22^ The optimisations were performed in the statistical software R^23^ using the package Alabama.^24^ To increase the likelihood that the diets would be acceptable to the population, each simulation minimised the deviation from the existing UK dietary pattern by minimising the sum of squared percentage differences from the current consumption, with the contributions from individual food groups weighted according to their price responsiveness and their share of the diet (reflecting a simplified measure of welfare—the degree to which people are unwilling to modify their consumption). All optimisation models were constrained to ensure that the resultant dietary patterns complied with the WHO recommendations and maintained the total calories and proportion of liquids in the diets. Simulations were performed to identify:

1. Optimised average dietary intake for the UK that met the WHO nutritional recommendations without any specification for GHG emissions reduction; and
2. Average dietary intake patterns optimised to achieve target reductions in dietary GHG emissions of 10%, 20%, 30%, 40%, 50% and 60% while meeting the WHO recommendations.^15^

### Health impact modelling

We modelled the impact on mortality in the UK associated with the modified dietary patterns resulting from changes in consumption of fruit, vegetables and red and processed meat. We conducted a literature search for meta-analyses relating food or nutrient consumption to non-communicable disease outcomes, and also used information published by the 2010 GBD study.^12^ The selected health outcomes were coronary heart disease, stroke, type 2 diabetes and cancers of the mouth/pharynx/larynx, oesophagus, lung, stomach and colon/rectum (table 1).^25–31^

**Table 1:** Dietary exposure-response pathways used in the health impact model

Dietary exposure	Health outcome	Relative risk (95% CI)	Source
Fruit	Coronary heart disease	0.93 (0.89 to 0.96) per 80 g increase per day	Dauchet *et al*^26^
	Stroke	0.89 (0.85 to 0.93) per 80 g increase per day	Dauchet *et al*^25^
	Oral cancer (mouth/pharynx/larynx)	0.72 (0.59 to 0.87) per 100 g increase per day	Marmot *et al*^31^
	Oesophagus cancer	0.56 (0.42 to 0.74) per 100 g increase per day	Marmot *et al*^31^
	Lung cancer	0.94 (0.90 to 0.97) per 80 g increase per day	Marmot *et al*^31^
	Stomach cancer	0.67 (0.59 to 0.76) per 100 g increase per day	Marmot *et al*^31^
Non-starchy vegetables	Coronary heart disease	0.89 (0.83 to 0.95) per 80 g increase per day	Dauchet *et al*^26^
	Stroke	0.97 (0.92 to 1.02) per 80 g increase per day	Dauchet *et al*^25^
	Oral cancer (mouth/pharynx/larynx)	0.72 (0.63 to 0.82) per 50 g increase per day	Marmot *et al*^31^
	Oesophagus cancer	0.87 (0.72 to 1.05) per 50 g increase per day	Marmot *et al*^31^
	Stomach cancer	0.70 (0.62 to 0.79) per 100 g increase per day	Marmot *et al*^31^
Red meat	Colorectal cancer	1.29 (1.04 to 1.60) per 100 g increase per day	Marmot *et al*^31^
	Type 2 diabetes	1.19 (1.04 to 1.37) per 100 g increase per day	Pan *et al*^28^
	Stroke	1.21 (1.10 to 1.33) per 100 g increase per day	Micha *et al*^29^
Processed meat	Colorectal cancer	1.21 (1.04 to 1.42) per 50 g increase per day	Marmot *et al*^31^
	Type 2 diabetes	1.51 (1.25 to 1.83) per 100 g increase per day	Pan *et al*^28^
	Coronary heart disease	1.37 (1.11 to 1.68) per 50 g increase per day	Micha *et al*^29^

The health impact calculations were performed using the life table model, IOMLIFET,^32^ implemented in R.^23^ Separate life tables were constructed for males and females, given their different underlying mortality rates. Age-specific and sex-specific data on population size, all-cause mortality and disease-specific mortality from the Office for National Statistics, General Register Office for Scotland and Northern Ireland Statistics and Research Agency were combined to create input data for the UK. To highlight the effects of the dietary modifications, we assume here that the diets are adopted instantly and underlying mortality rates remain constant for the duration of follow-up. For the analysis, the exposure–response functions were assumed to be log-linear and, in cases where several dietary exposures affected the same disease, the risks were multiplied together. The model outputs were changes in years of life lost (YLL) for the UK population over 20 and 30 years.

To account for time lags between dietary changes and changes in health outcomes, time-varying functions based on cumulative distribution functions of normally distributed variables (s-shaped curves) were used. The shapes of the functions were informed by empirical evidence of the effects of dietary interventions on various causes of mortality over time.^33–36^ The assumed lags for coronary heart disease, stroke and type 2 diabetes reach a maximum impact after approximately 10 years and for cancers after 30 years, with no change in cancer risk for the first 10 years (see web materials).

We assessed the sensitivity of the health impact model results in two areas:

1. To test the sensitivity of the results to key parameters, we generated upper and lower health impact estimates using high and low estimates for GHG emissions for each food group based on evidence from the literature, combined with high and low estimates for the relative risks based on published 95% CIs from the source meta-analyses (table 1). These simulations were performed for a 20% GHG reduction only.
2. Since evidence of the independence of health effects due to fruit and vegetable consumption is currently unclear,^37^ including both may lead to double counting. There is also evidence that relative risks linking vegetable consumption and coronary heart disease may be overestimated.^26^ As a ‘structural test’ of the model, we therefore repeated the main analysis including effects due to (i) vegetables but not fruit and (ii) fruit but not vegetables.

Further details of the methods are contained in online supplementary web materials.

## Results

According to our optimisation model, in order to conform to the WHO nutritional recommendations, the UK diets would need to contain less red meat, dairy products, eggs and sweet and savoury snacks, but more cereals, fruit and vegetables.^15^ Modelled changes for all food groups can be found in the online supplementary web materials. Since the required changes to average male diets are greater than those for females, the resulting dietary exposure changes are correspondingly greater (table 2). When no dietary GHG reduction is required (the diet is optimised solely to meet the WHO guidelines), there is a large increase in consumption of fruit and a somewhat smaller increase in consumption of vegetables. However, as GHG emissions are reduced, there is a shift in the optimised diets, with increasingly more vegetables and less fruit (though still more than in current diets). As emissions are progressively reduced, consumption of red and processed meats in the optimised diets is also reduced and eventually removed altogether.

**Table 2:** Modelled changes in average dietary intakes in the UK (relative to current diets) for different levels of GHG reduction

		Modelled change (and % change) from current average diet (g/day)							
GHG reduction		Fruit		Non-starchy vegetables		Red meat		Processed meat	
Target (%)	Achieved (%)	Males	Females	Males	Females	Males	Females	Males	Females
0*	17.2	+110.2	+96.8	+53.3	+57.0	−16.2	−4.5	−37.4	−3.2
		(+80.4%)	(+66.7%)	(+53.6%)	(+56.4%)	(−38.0%)	(−15.8%)	(−63.0%)	(−8.8%)
10	18.0	+109.0	+94.9	+54.5	+58.9	−16.1	−3.4	−38.0	−3.8
		(+79.5%)	(+65.4%)	(+54.9%)	(+58.3%)	(−37.8%)	(−12.1%)	(−64.1%)	(−10.5%)
20	21.9	+109.4	+97.2	+54.2	+56.6	−17.1	−11.7	−36.2	−5.9
		(+79.8%)	(+67.0%)	(+54.5%)	(+56.0%)	(−40.0%)	(−41.3%)	(−61.0%)	(−16.1%)
30	30.0	+107.1	+86.1	+56.4	+67.8	−27.2	−21.7	−37.7	−5.9
		(+78.2%)	(+59.3%)	(+56.7%)	(+67.0%)	(−63.9%)	(−76.7%)	(−63.6%)	(−16.4%)
40	40.0	+95.4	+77.6	+68.2	+76.2	−35.6	−24.8	−43.0	−11.5
		(+69.6%)	(+53.5%)	(+68.6%)	(+75.4%)	(−83.6%)	(−87.4%)	(−72.5%)	(−31.7%)
50	50.0	+84.6	+68.9	+78.9	+84.9	−42.6	−27.0	−56.0	−23.2
		(+61.7%)	(+47.5%)	(+79.4%)	(+84.0%)	(−100.0%)	(−95.5%)	(−94.4%)	(−64.0%)
60	60.0	+36.7	+45.2	+126.9	+108.6	−42.6	−28.3	−59.3	−36.3
		(+26.7%)	(+31.2%)	(+127.6%)	(+107.4%)	(−100.0%)	(−100.0%)	(−100.0%)	(−100.0%)

*Diet optimised to achieve the WHO guidelines with no GHG reduction target.

GHG, greenhouse gases.

Translation of the optimised dietary patterns into estimated health impacts shows that even diets not constrained to reduce GHG emissions can result in significant beneficial effects on health since the new diets meet the WHO recommendations (table 3). This scenario, with no GHG reduction target, produced an incidental reduction in emissions of over 17%. Although this is short of the needed reduction in emissions from the food and agriculture sector as suggested by the UK Climate Change Committee,^1^ our model suggests that the optimised diets would result in a saving of more than 6.8 million YLL over 30 years. This would represent a gain of 12 months of life expectancy for the current birth cohort of males and more than 4 months for females (approximately 8 months on average). Around 70% of this impact is from coronary heart disease and there is also substantial benefit for stroke. Cancer benefits would be likely to be relatively modest initially (due to the initial latency period), but these would become more significant over the longer term, as demonstrated by the impacts over 30 years.

**Table 3:** Modelled health impacts (cumulative reduction in Years of Life Lost, YLL) associated with dietary changes in the UK over 20 and 30 years for a scenario with no GHG reduction target

Health outcome	Cumulative reduction in YLL*	
	Over 20 years	Over 30 years
Coronary heart disease	2 098 200	4 810 400
Stroke	428 000	947 700
Oral cancer	14 600	136 400
Oesophageal cancer	33 900	313 100
Lung cancer	26 600	247 600
Stomach cancer	22 100	200 600
Colorectal cancer	15 900	144 600
Type 2 diabetes	18 900	42 400
Total	2 658 200	6 842 800

*Figures rounded to the nearest 100.

**Figure 1:**
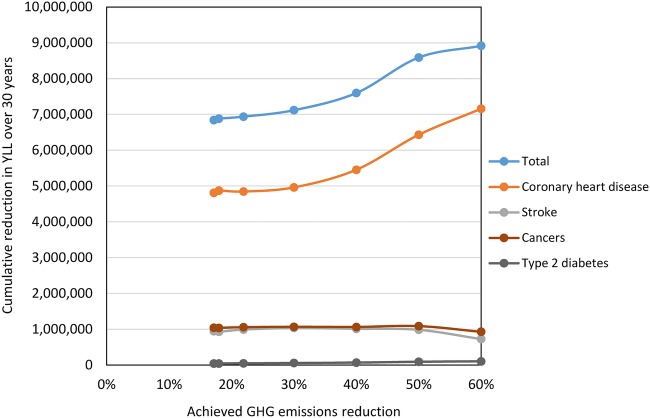
Modelled health impacts associated with dietary changes in the UK for different levels of greenhouse gases (GHG) reduction.

For simulations requiring incremental increases in the level of required GHG emissions mitigation, our results suggest that reducing emissions would lead to a net benefit for health which increases until the emissions are reduced radically, at which point the health benefits may decline (figure 1). In particular, benefits for stroke and cancers peak at a GHG reduction of 50% and are lower for greater reductions. While the 60% GHG reduction still results in large savings of over 8.9 million YLL (30 years), the diet only barely meets the WHO guidelines and its composition is limited to a relatively few food groups. There may also be adverse effects due to reduced consumption of calcium and vitamins (see web materials).

**Figure 2:**
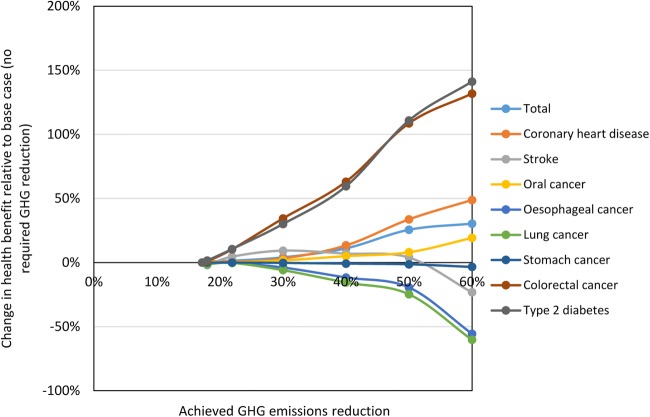
Relative changes in modelled health impacts for incremental increases in greenhouse gases (GHG) reduction target.

Figure 2 shows the additional health benefits for each outcome as the achieved GHG reduction increases relative to the optimised diet in which no GHG reduction is required. Again, it can be seen that the total health benefit increases as GHG emissions are reduced. However, the additional impact at 60% GHG reduction is only 30% greater than that achieved without a GHG reduction target. In addition, benefits for stomach cancer, oesophageal cancer and lung cancer decrease consistently as GHG emissions are reduced due to reductions in fruit consumption.

### Sensitivity analysis

**Figure 3:**
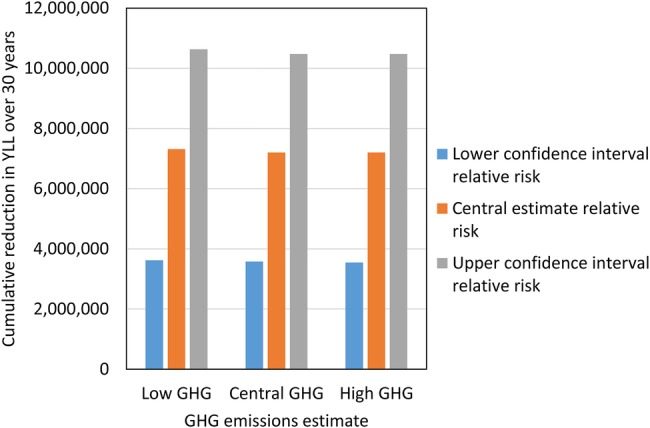
Sensitivity of modelled health impacts (20% greenhouse gases (GHG) reduction) to low/high estimates of GHG emissions and exposure–response functions.

Sensitivity analysis demonstrates that the majority of the uncertainty relates to the exposure–response functions rather than the GHG emissions (figure 3). The results provide an indication of the ranges around our central model estimates. Since the impacts were modelled using relative risks based on published meta-analyses, the central exposure–response estimates are most likely to be closest to the ‘true’ associations.

**Figure 4:**
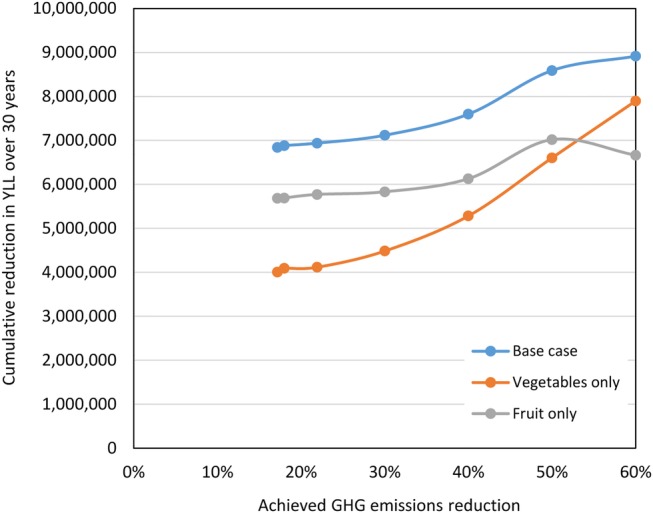
Sensitivity of modelled health impacts to inclusion of effects due to fruit and vegetables for different levels of greenhouse gases (GHG) reduction.

Analyses which accounted only for impacts on health due to consumption of either vegetables *or* fruit reduced the total impacts by around 41% (vegetables only) and 17% (fruit only) for the scenario with no GHG reduction target. As the emissions are progressively reduced, the optimal balance of total fruit and vegetable consumption gradually shifts towards increasing vegetable consumption, since vegetables are associated with lower GHG emissions than fruits on average (figure 4). Counting only impacts due to vegetables therefore leads to additional increases in benefits as GHG emissions are reduced. On the other hand, only counting fruit reduces the benefits for several cancers at greater levels of GHG reduction.

## Discussion

Our results show that substantial benefits for health and climate change mitigation can be achieved in the UK by modifying existing diets so that they meet nutritional requirements while also reducing GHG emissions. We have also demonstrated that this can be achieved in ways which maintain the likely acceptability of diets for emission reductions up to 40%. The new diets would contain fewer animal products and savoury snacks and more fruit, vegetables and cereals. Even diets requiring no reduction in GHG emissions were found to result in an incidental reduction of over 17%. Our model suggests that such changes to the UK diets would save almost 7 million YLL over 30 years and increase life expectancy at birth by around 8 months, primarily from benefits to coronary heart disease. Additional health benefits were found to accrue as the GHG emissions associated with diets were progressively reduced. However, the health gains of incremental emission reductions would be lower than those obtained purely by optimising diets to meet health targets alone. Furthermore, if emissions are reduced radically, the optimised diets favour consumption of vegetables over consumption of fruit, since emissions associated with vegetables are lower on average. Therefore, benefits for some health outcomes may begin to reduce and the overall health benefit appears to reach a plateau, driven primarily by the fact that there is no more avoidable meat in the diet.

In general, the level of deviation in the diets from current diets (% deviation from the current diet after normalisation by price elasticities and food expenditure shares) remained low and relatively constant when dietary GHG emissions were reduced by 40% or less. However, as emissions were reduced further, the deviation from the current diet increased dramatically, suggesting that diets in which emissions are reduced by more than 40% are unlikely to be acceptable in the UK unless preferences change in the future.^15^ This suggests a natural limit to how much can be achieved for health and climate change mitigation by dietary change alone.

Previous studies have attempted to quantify GHG emission reductions associated with dietary changes using similar optimisation methods to ours.^38^ However, our study has used a novel application of an optimisation approach to model the detailed composition of the entire diet and attempted to maintain its acceptability implicitly through incorporation of information on consumer behaviour (price elasticity and expenditure share). Our method therefore has the considerable advantage of generating more realistic, detailed diets for the UK. This does not, however, necessarily ensure that our dietary scenarios would be acceptable for consumption. In those few studies where the associated health impacts were estimated, the analysis is usually based on hypothetical scenarios in which the nutritional composition of the diet is adjusted to meet dietary or GHG goals.^4^ ^5^ ^8^ ^9^ In all cases, these hypothetical scenarios were shown to lead to considerable benefits for health and GHG reduction.

The limitations of the study relate primarily to inadequacies of the available input data and some of the assumptions (as with all modelling analyses). It is most likely that food consumption reported in the NDNS is an underestimate of actual intake but this will not affect the relative contributions of different food groups.^39–41^ The NDNS is the most recent data set available for the UK and almost certainly the most accurate. For GHG emissions, we used complete LCI emissions specific to the UK where possible. Our estimates are likely to be somewhat different from other estimates since they incorporate emissions from production, processing, packaging, transport, storage and waste, which are often not included. New advice from the United Nations’ Intergovernmental Panel on Climate Change Working Group 1 suggests that different climate active pollutants and GHG should not be combined to produce a single carbon dioxide equivalent (CO_2_e) measure of relative climate forcing because they have different effects over a range of time periods.^42^ However, the literature to date largely follows earlier convention.

We selected a relatively limited subset of health outcomes for modelling despite evidence of dietary associations for other outcomes.^12^ ^30^ In general, epidemiological relationships reported in the literature are not adjusted for consumption of other foods, so we purposely limited the range of modelled outcomes to avoid double counting. We also did not take into account relationships between saturated and unsaturated fat consumption and coronary heart disease because it would be likely to be confounded by the (modelled) relationship between meat consumption and coronary heart disease. We have modelled impacts only on mortality, whereas actual benefits to health would be considerably greater were corresponding impacts on morbidity also included. We also used current mortality rates, although these are declining and may continue to do so in the future (similarly for cardiovascular disease). However, future trends are unclear, for example, because of increases in obesity.^43^ Ultimately, our estimates should be treated as indicative of broader patterns rather than precise estimates of the total potential impact.

This study has shown that substantial health benefits could be achieved in the UK by making relatively modest dietary changes which would also benefit the environment. These cobenefits are largely achieved by reducing the consumption of animal products (and switching away from high-emission meats such as beef and lamb and towards lower emission meats such as pork and chicken) and savoury processed foods, while increasing consumption of cereals, fruit and vegetables. However, the health benefits and acceptability of such diets is likely to peak at around a 40% reduction in GHG emissions—greater reductions than this would be likely to result in unacceptable diets and progressively reduced health gains (though still improved relative to current diets). Consequently, more dramatic emission reductions may be required from other sectors of society.

The results show that radical dietary changes such as veganism are not necessary in order for there to be large reductions in GHG emissions and quantifiable benefits to health. Indeed, making dietary changes that are too restrictive in terms of emissions reduction is likely to place limits on the health benefits that can be achieved by restricting consumption of some healthy foods (such as fruit). Instead, encouraging people in the UK to modify their diets to contain fewer animal products and processed foods and more cereals, fruit and vegetables would produce tangible benefits to both health and the environment.

## References

[1] CCC. Building a low-carbon economy—the UK's contribution to tackling climate change. London: Committee on Climate Change, 2008.

[2] Audsley E, Brander M, Chatterton J How low can we go? An assessment of greenhouse gas emissions from the UK food system and the scope for reduction by 2050. WWF-UK, 2009.

[3] Stehfest E, Bouwman L, van Vuuren DP Climate benefits of changing diet. Clim Change 2009;95:83–102. 10.1007/s10584-008-9534-6

[4] Scarborough P, Allender S, Clarke D Modelling the health impact of environmentally sustainable dietary scenarios in the UK. Eur J Clin Nutr 2012;66:710–15. 10.1038/ejcn.2012.34 22491494 PMC3389618

[5] Friel S, Dangour AD, Garnett T Public health benefits of strategies to reduce greenhouse-gas emissions: food and agriculture. Lancet 2009;374:2016–25. 10.1016/S0140-6736(09)61753-0 19942280

[6] Berners-Lee M, Hoolohan C, Cammack H The relative greenhouse gas impacts of realistic dietary choices. Energ Policy 2012;43:184–90. 10.1016/j.enpol.2011.12.054

[7] Macdiarmid JI, Kyle J, Horgan GW Sustainable diets for the future: can we contribute to reducing greenhouse gas emissions by eating a healthy diet? Am J Clin Nutr 2012;96:632–9. 10.3945/ajcn.112.038729 22854399

[8] Scarborough P, Nnoaham KE, Clarke D Modelling the impact of a healthy diet on cardiovascular disease and cancer mortality. J Epidemiol Community Health 2012;66:420–6. 10.1136/jech.2010.114520 21172796

[9] Aston LM, Smith JM, Powles JW Impact of a reduced red and processed meat dietary pattern on disease risks and greenhouse gas emissions in the UK: a modelling study. BMJ Open 2012;2:e001072 10.1136/bmjopen-2012-001072 PMC3467613 22964113

[10] Tukker A, Goldbohm RA, de Koning A Environmental impacts of changes to healthier diets in Europe. Ecol Econ 2011;70:1776–88. 10.1016/j.ecolecon.2011.05.001

[11] Capacci S, Mazzocchi M, Shankar B Policies to promote healthy eating in Europe: a structured review of policies and their effectiveness. Nutr Rev 2012;70:188–200. 10.1111/j.1753-4887.2011.00442.x 22364161

[12] Lim SS, Vos T, Flaxman AD A comparative risk assessment of burden of disease and injury attributable to 67 risk factors and risk factor clusters in 21 regions, 1990–2010: a systematic analysis for the Global Burden of Disease Study 2010. Lancet 2012;380:2224–60. 10.1016/S0140-6736(12)61766-8 23245609 PMC4156511

[13] Bates B, Lennox A, Prentice A National Diet and Nutrition Survey: Headline results from Years 1, 2 and 3 (combined) of the Rolling Programme (2008/2009–2010/11). London: Department of Health, Food Standards Agency and NatCen Social Research, 2012.

[14] Rayner M, Scarborough P The burden of food related ill health in the UK. J Epidemiol Community Health 2005;59:1054–7. 10.1136/jech.2005.036491 16286493 PMC1732963

[15] Green R, Milner J, Dangour AD UK food-based greenhouse gas emissions are reduced through realistic and healthy dietary change. Climatic Change 2015;129:253–65.

[16] Weiss F, Leip A Greenhouse gas emissions from the EU livestock sector: a life cycle assessment carried out with the CAPRI model. Agric Ecosyst Environ 2012;149:124–34. 10.1016/j.agee.2011.12.015

[17] Vieux F, Darmon N, Touazi D Greenhouse gas emissions of self-selected individual diets in France: changing the diet structure or consuming less? Ecol Econ 2012;75:91–101. 10.1016/j.ecolecon.2012.01.003

[18] Hammerschlag K, Venkat K Meat-eater's guide to climate change and health: lifecycle assessments—methodology and results. Washington, DC, USA: Environmental Working Group, 2011.

[19] Garnett T Where are the best opportunities for reducing greenhouse gas emissions in the food system (including the food chain)? Food Policy 2011;36:S23–32. 10.1016/j.foodpol.2010.10.010

[20] Foster C, Green K, Bleda M Environmental impacts of food production and consumption: a report to the department for environment, food and rural affairs. Defra, London, UK: Manchester Business School, 2006.

[21] Venkat K The climate change and economic impacts of food waste in the United States. Int J Food System Dynamics 2011;2:431–46.

[22] WHO. Diet, Nutrition, and the Prevention of Chronic Disease: Report of a Joint WHO/FAO Expert Consultation. Geneva, Switzerland: World Health Organization, 2003, Contract No.: WHO Technical Report Series 916.

[23] R Core Team. R: A language and environment for statistical computing. Vienna, Austria: R Foundation for Statistical Computing Contract No.: ISBN 3-900051-07-0. http://www.R-project.org/

[24] Varadhan R Alabama: Constrained nonlinear optimization. 2012. http://CRAN.R-project.org/package=alabama

[25] Dauchet L, Amouyel P, Dallongeville J Fruit and vegetable consumption and risk of stroke: a meta-analysis of cohort studies. Neurology 2005;65:1193–7. 10.1212/01.wnl.0000180600.09719.53 16247045

[26] Dauchet L, Amouyel P, Hercberg S Fruit and vegetable consumption and risk of coronary heart disease: a meta-analysis of cohort studies. J Nutr 2006;136:2588–93. 16988131 10.1093/jn/136.10.2588

[27] WCRF. Food, nutrition, physical activity, and the prevention of cancer: a global perspective. Washington, DC: World Cancer Research Fund/American Institute for Cancer Research, 2007.

[28] Pan A, Sun Q, Bernstein AM Red meat consumption and risk of type 2 diabetes: 3 cohorts of US adults and an updated meta-analysis. Am J Clin Nutr 2011;94:1088–96. 10.3945/ajcn.111.018978 21831992 PMC3173026

[29] Micha R, Wallace SK, Mozaffarian D Red and processed meat consumption and risk of incident coronary heart disease, stroke and dibaetes mellitus—a systematic review. Circulation 2010;121:2271–83. 10.1161/CIRCULATIONAHA.109.924977 20479151 PMC2885952

[30] Kelly JH, Sabate J Nuts and coronary heart disease: an epidemiological perspective. Brit J Nutr 2006;96:S61–7. 10.1017/BJN20061865 17125535

[31] Marmot M, Atinmo T, Byers T Food, nutrition, physical activity, and the prevention of cancer: a global perspective. World Cancer Res Fund/Am Inst Cancer 2007;46:312–14.

[32] Miller B, Hurley J Life table methods for quantitative impact assessments in chronic mortality. J Epidemiol Community Health 2003;57:200–6. 10.1136/jech.57.3.200 12594196 PMC1732393

[33] Capewell S, O'Flaherty M Can dietary changes rapidly decrease cardiovascular mortality rates? Eur Heart J 2011;32:1187–9. 10.1093/eurheartj/ehr049 21367835

[34] Franco M, Ordunez P, Caballero B Impact of energy intake, physical activity, and population-wide weight loss on cardiovascular disease and diabetes mortality in Cuba, 1980–2005. Am J Epidemiol 2007;166:1377–80. 10.1093/aje/kwm226 17881386

[35] Harashima E, Nakagawa Y, Urata G Time-lag estimate between dietary intake and breast cancer mortality in Japan. Asia Pac J Clin Nutr 2007;16:193–8. 17215198

[36] Tsuji K, Harashima E, Nakagawa Y Time-lag effects of dietary fiber and fat intake ratio on Japanese colon cancer mortality. Biomed Environ Sci 1996;9:223–8. 8886335

[37] He FJ, Nowson CA, Lucas M Increased consumption of fruit and vegetables is related to a reduced risk of coronary heart disease: meta-analysis of cohort studies. J Hum Hypertens 2007;21:717–28. 10.1038/sj.jhh.1002212 17443205

[38] Wilson N, Nghiem N, Ni Mhurchu C Foods and dietary patterns that are healthy, low-cost, and environmentally sustainable: a case study of optimization modeling for New Zealand. PLoS ONE 2013;8:e59648 10.1371/journal.pone.0059648 23544082 PMC3609827

[39] Macdiarmid JI, Blundell J Assessing dietary intake: who, what and why of under-reporting. Nutr Res Rev 1998;11:231–53. 10.1079/NRR19980017 19094249

[40] Hill RJ, Davies PSW The validity of self-reported energy intake as determined using the doubly labelled water technique. Brit J Nutr 2001;85:415–30. 10.1079/BJN2000281 11348556

[41] Black AE The sensitivity and specificity of the Goldberg cut-off for EI:BMR for identifying diet reports of poor validity. Eur J Clin Nutr 2000;54:395–404. 10.1038/sj.ejcn.1600971 10822286

[42] IPCC. Climate Change 2013: The Physical Science Basis. IPCC Working Group I Contribution to Fifth Assessment Report (AR5). Intergovernmental Panel on Climate Change, 2013.

[43] Nichols M, Townsend N, Scarborough P Trends in age-specific coronary heart disease mortality in the European Union over three decades: 1980–2009. Eur Heart J 2013;34:3017–27. 10.1093/eurheartj/eht159 23801825 PMC3796269

